# Fluoride ingestion and thyroid function in children resident of naturally fluoridated areas - An observational study

**DOI:** 10.4317/jced.55812

**Published:** 2019-10-01

**Authors:** Naseemoon Shaik, Raghavendra Shanbhog, B Nandlal, H.M Tippeswamy

**Affiliations:** 1 Assistant professor, Department of Pediatric Dentistry, MNR Dental College and Hospital, Sangareddy, Hyderabad; 2 Reader, Department of Pediatric Dentistry, JSS Dental College and Hospital, Mysuru; 3 Professor, Department of Pediatric Dentistry, JSS Dental College and Hospital, Mysuru; 4 Reader, Department of public health dentistry, JSS Dental College and Hospital, Mysuru

## Abstract

**Background:**

Literature shows association between systemic fluorides with water fluoride level above 3ppm and endocrine disorders especially related to thyroid. Aim & Objectives: To estimate serum T3, T4, TSH, Fluoride levels among children with normal nutritional status and optimal iodine intake, residing in three different ranges of drinking water fluoride levels below 3ppm.

**Material and Methods:**

Present double blinded observational trial comprised of 293 children aged between 9-13 years consuming naturally fluoridated water of 3 different ranges: Group I: 0.01 - 0.6ppm, Group II: 0.7-1.2ppm and Group III: 1.3-1.8ppm. For each child demographic data, BMI and Clinical Fluorosis Index were recorded along with serum T3, T4, TSH, Fluoride levels. Data was analyzed using Chi Square, Kruskal Wallis test and Repeated measures of ANOVA with SPSS 23.

**Results:**

For serum TSH levels 40% of children of group I had deranged levels followed by group III (20%) and Group II (16%). For serum T4 levels 24% of children of both groups I and III had deranged levels followed by group II (20%). Inter group correlation of drinking water fluoride levels to number of deranged serum T3, T4, and TSH of the children showed non-significant association.

**Conclusions:**

According to the present study results long term intake of fluoridated drinking water (0.02 -1.4 ppm) did not showed effect on the thyroid function in the children with normal nutritional status and optimal iodine intake.

** Key words:**Iodine, nutrition, serum fluoride, systemic fluoride, thyroid function test.

## Introduction

Fluoridation of community water supplies is the defined adjustment of fluoride levels in drinking water to an optimal level for the prevention of dental decay. Studies conducted in the past 60 years across the worldhave consistently indicated that fluoridation of community water supplies is effective in preventing dental decay in both children and adults. Centers for Disease Control and Prevention stated that community water fluoridation as one of 10 great public health achievements of the 20th century (1). Despite its merits, community water fluoridation is a controversial public health intervention. Allegations include increased overall mortality, occurrence of Down syndrome, Specific cancers, endocrine disorders, behavioral, cognitive and other neurological ill effects (1).

Thyroid diseases are one of the commonest endocrine disorders worldwide including India with about 42 million people suffering from it (2). Considering a potential association between fluoride exposure to endocrine disruption especially thyroid, many in-vitro experimental, animals and human studies have been published with more concern on thyroid (3-12). But there exists a lack of clarity. Few studies reported that excessive long-term intake of fluoride, is a significant risk factor for the development of thyroid dysfunction (5-7). One study in 1999 reported significant reduction in serum thyroxin (T4) with increased levels of triiodothyronine (T3); thyroid-stimulating hormone (TSH) (11). Another study in 2001 reported T3 and T4 concentrations in the serum of the patients with endemic fluorosis were significantly below the normal reference value (12). In-contradiction few studies have indicated that the high fluoride intake does not have any effect on thyroid function (8-10).

British Fluoridation Society (2006) based on the Royal College of Physicians, York Systematic committee and World Health Organization IPCS review stated that “there is no evidence that fluoride is responsible for any disorder of the thyroid when consumed in optimal level”. The “optimal level” has been under revision through various authentic bodies over the years. Recently, the Department of Health and Human Services (HHS) issued proposed recommendations to change the recommended optimal amount of fluoride in drinking water from 0.7 to 1.2 mg/L based on ambient air temperature to a uniform amount of 0.7 mg/L. Introduction of different fluoride delivery sources resulted in an uncoordinated delivery of fluoride in optimal doses especially in naturally fluoridated areas (13).

The research hypothesis was long term consumption of fluoridated drinking water at optimum or little above optimal level affect the thyroid function in children with normal nutritional status and optimum iodine intake. The present study was carried out with the objective to estimate serum T3, T4, TSH, Fluoride levels among children with normal nutritional status and optimal iodine intake residing in three different ranges of drinking water fluoride levels.

## Material and Methods

-Study design and ethical clearance

The study design was a double blinded observational trial. The study plan was approved by the institutional ethical committee. All information about the children and their identity was anonymous. After giving written information about the nature of the study, permission for selecting the samples was obtained from concerned government and school authorities. Participants and their parents were explained about the nature of the study and written informed consent was obtained. They were informed to withdraw from the study at any point of time during the observational trial.

-Study area selection

The children for present study were selected from villages of Mysore Taluk with the necessary drinking water fluoride level. Based on drinking water fluoride level reports of 2011(District level water quality testing laboratory), 212 naturally fluoridated villages from 35 Grampanchayaths of Mysore, were segregated into three categories as follows; below optimal 0.01 to 0.6ppm, optimal 0.7 to 1.2ppm and slightly above optimal 1.3 to 1.8 ppm. From each category 15 villages were randomly selected for drinking water fluoride analysis.

-Collection and analysis of water samples

From each selected village, after discussion with local in-charge person for water supply major drinking water sources were identified. From the identified source 500 ml of water sample was collected for analysis as per APHA (1998) guidelines in sterile HDPE bottles. The collected samples were labeled and transported to lab for further analyses. Water analysis was carried out in research unit, according to analytical protocol using OAKTON Fluoride Ion Selective Electrode Equipment, USA. Each sample was analyzed twice for confirmation. The analyzed drinking water fluoride concentration of each village was tabulated. Of the 45 villages analyzed for drinking water fluoride levels, the 26 villages showing variation in fluoride levels from that of 2011 report were excluded because the present study focused on long term effects of fluoride. The selected 19 villages in the study were (6 villages with water fluoride level 0.01 to 0.6ppm, 8- with villages with water fluoride level 0.7 to 1.2ppm and 5 villages with water fluoride level 1.3 to 1.8ppm).

-Sample size calculation 

Sample size was calculated based on previous study results indicating 54.4% subjects consuming fluoridated drinking water had deranged hormonal levels. Using 90% of power and 95% confidence interval the sample size was found to be 90. (N MASTER SAMPLE SIZE CALCULATING SOFTWARE)Final sample size 270, n = 90 per each group.

-Subject selection

From 19 identified villages, all available 1036 school going children between the ages 9-13 years were screened for selection criteria.Healthy childrenwith good general health, lifelong residents of their particular locality, normal nutritional status,consuming Iodized salt and who is willing to participate in the study and give written informed consent were included. Demographic information of the childrenwas collected from the school records. Information regarding town of residence, source of water use from birth to age three, current water source for both cooking and drinking was collected by interviewing the parents. The adequacy of the childrennutritional status was accessed through IAP Growth Charts (Revised IAP Growth Charts for Height, Weight and Body Mass Index for 5- to 18-year-old Indian Children - 2015) (14) for which anthropometric measurements of child were collected by weighing the child on a calibrated load cell operated electronic scale accurate to 50g while they were dressed in light clothing without shoes, and measuring body heights with a stature meter containing a metal tape accurate to 0.1mm. The childrennot fulfilling selection criteria were excluded to obtain 293 childrenfrom whom the samples for analysis were collected. Childrenwere targeted for high, expected levels of cooperation and low population mobility. Childrenwere selected with the view of recruiting populations at varying levels of fluoride exposure. Childrenwere selected as per three population groups exposed to a range of water fluoride content: Group I: 0.01 - 0.6ppm- 98 children, Group II: 0.7-1.2ppm-103 children and Group III: 1.3-2ppm-92.

-Clinical examination and blood sample

A trained pediatric dentist carried out clinical examination to assess Dental fluorosis. For each subject fasting venous blood samples (5 ml) from Dorsal Hand Vein, as per protocol was collected by trained & experienced para-medical staff. The collected blood samples were transferred to pediatric vacutainer tubes, gently inverted and labeled for Patient’s name, age, Medical record number and date. Collected Blood was preserved in clean plastic centrifuge tubes and immediately centrifuged for 10 min at 6000 rpm. Serum was quickly removed to other clean plastic tubes and was kept in a refrigerator at -40°C until the analysis.

-Analysis of blood sample:

The level of serum T3, T4 was determined with Competitive Chemi Luminescent Immunoassay kits and level of serum TSH was determined with ULTRA SENSITIVE SANDWICH CHEMI LUMINESCENT IMMUNO ASSAY with analyzer according to the manufacturer recommendation. The collected data was tabulated and statistically analyzed. All the statistical operations were done through SPSS (Statistical Presentation System Software) (version 23.0). Data analysis was performed using paired t test and repeated measures of ANOVA.

## Results

Stratification of children according to gender across study groups with their mean age; Mean drinking water fluoride levels, mean serum fluoride level and prevalence of dental fluorosis was tabulated in  Table 1. The mean age of selected children fell in the range of 10.1to 10.8 years with varied distribution of gender across the group. Drinking water fluoride level in group I, II and III fell in the range of 0.12 to 0.32 (mean of 0.22.), 0.76 to 1.1(mean of 0.83) and 1.43-1.46 (mean of 1.44.) respectively. Mean serum fluoride levels and prevalence of dental fluorosis in children increased with the increase in drinking water fluoride levels. Dental fluorosis evaluated through clinical Dean’s fluorosis index across the groups showed maximum prevalence in group III (88%) followed by group II (45%) and least in Group I (4%).

**Table 1:**
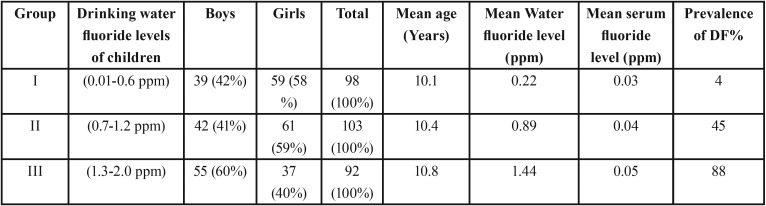
Stratification ofchildrenaccording to gender across study groups with mean age; Mean drinking water fluoride levels, mean serum fluoride level and prevalence of dental fluorosis across study groups.

Among 293 children evaluated for thyroid function consuming different levels of fluoride through drinking water, 66 (22.5%) children showed deranged levels. In group I (Drinking water fluoride levels 0.01 to 0.6 ppm) of 98 children evaluated for thyroid function 21 (40%) children showed derangement in Serum TSH levels and 24 (24%) children showed derangement in Serum T4 levels. No child exhibited derangement in Serum T3 levels 

In group II (Drinking water fluoride levels 0.07 to 1.2 ppm) of 103 children evaluated for thyroid function 16(16%) children showed derangement in Serum TSH levels and 21 (20%)children showed derangement in Serum T4 levels. No child exhibited derangement in Serum T3 levels.

In group III (Drinking water fluoride levels 1.3 to 1.8 ppm) of 92 children evaluated for thyroid function 18 (20%) children showed derangement in Serum TSH levels and 22 (24%) children showed derangement in Serum T4 levels. No child exhibited derangement in Serum T3 levels.

Even though group I showed more children with TSH derangement than in other groups inter group correlation between drinking water fluorides to deranged serum TSH cases showed non-significant association. All the children with deranged TSH levels showed values falling between 4.5–10 mU/liter indicating children having subclinical hypothyroidism. Inter group correlation for drinking water fluoride levels to number of deranged T4 children by Chi-square test and Kruskal Wallis Test showed non-significant association ( Table 2, Table 3).

**Table 2:**
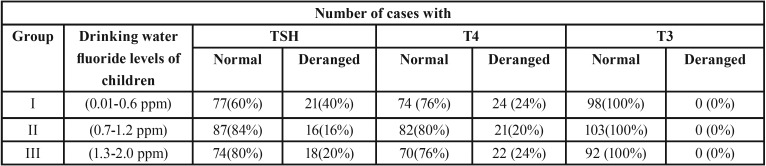
Distribution ofstudy subjects according to Serum TSH, T3 and T4 levels across the study group.

**Table 3:**
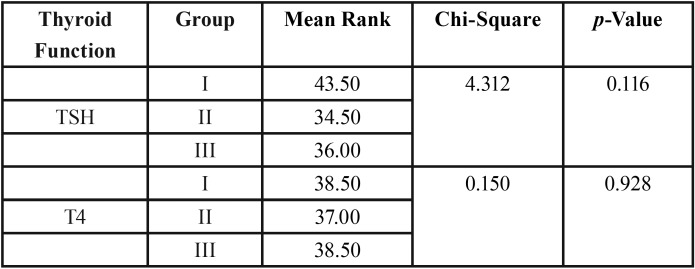
Serum TSH and T4 levels-Inter group comparison.

Comparing mean TSH levels group I showed highest value (3.86) followed by Group III (3.27) and Group II (3.21). Comparing mean T4 levels group I showed highest value (9.53) followed by Group III (9.29) and Group II (8.79). Comparing mean T3 levels group III showed highest value (158.36) followed by Group II (156.84) and Group I (140.80).Intergroup comparison for drinking water fluoride levels to mean serum TSH, T3 and T4 levels with ANOVA showed non-significant correlation ( Table 4).

**Table 4:**
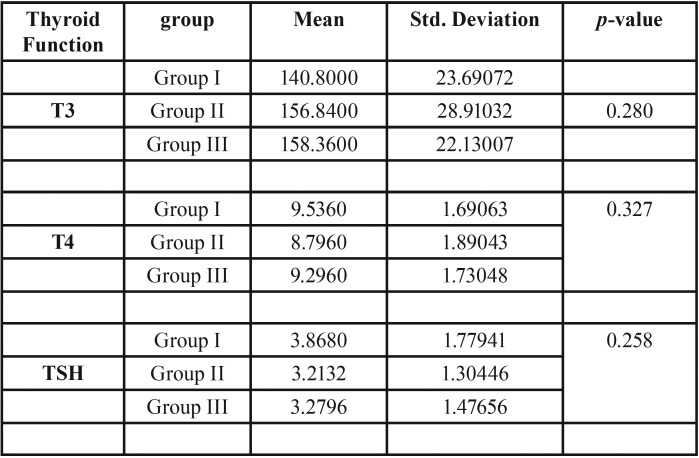
Mean Thyroid hormone levels across the Study group.

## Discussion

Across the globe approximately 25 countries are having artificial water fluoridation to varying levels and about 435 million people worldwide receive water fluoridated at the recommended level. 28 countries have water that is naturally fluoridated, though in many of them the fluoride is above the recommended safe level. India which falls under naturally occurring fluoride region with more than 20 states endemic for dental fluorosis with 66 million people consuming naturally fluoridated water with varied concentration. The recommended optimal fluoride level in drinking water is changed from 0.7 to 1.2 mg/L based on ambient air temperature to a uniform amount of 0.7 mg/L. However according to the Bureau of Indian Standards 2009 Second Revision IS 10500, the permissible limit of fluoride in drinking water in the absence of alternate source is 1.5ppm.

Thyroid diseases are the most common endocrine disorders worldwide including India. In India every seventh person is suffering from a thyroid disorder (15). Literature reports a potential association between fluoride exposures to endocrine disruption, especially thyroid. Hence it is of prime importance to address this issue (5, 6, and 7).

The present study was carried out with the objective to estimate serum Thyroid function among children with normal nutritional status and optimal iodine intake residing in three different ranges of drinking water fluoride levels. It showed non-significant association between thyroid function and long term systemic intake of fluoride up to 1.4ppm. The present study results shown a non-significant association between dental fluorosis to thyroid function in children residing in near optimal naturally fluoridated areas (0.2- 1.4ppm) with normal nutritional status and optimal iodine intake.

In accordance to our study results Baum K 1981 (16) and Barberio *et al.*, 2017 (17) reported fluoride exposure do not increase susceptibility to impaired thyroid functioning at the population-level. Our results are also in accordance with the endorsed statement quote by the British Thyroid association in 2006 and European Union’s 2011 SCHER report on health and environmental risks of fluoride, that human studies do not suggest an association between water fluoridation and any thyroid disorder.

In contradiction to our results, studies by Wilson *et al.*, 1941 (18), Murray *et al.*, (1948) (19), Steyn *et al.*, (1955) (20), Obel *et al.*, (1982) (21), Jooste *et al.*, (1999) (22), Rathee *et al.*, (2004) (23), Susheela *et al.*, (2005) (4), Xiang *et al.*, (2009) (24) , Peckham *et al.*, 2015 (25), Sachdeva *et al.*, (2015) (26). found evidence of at least one or more thyroid hormone derangement among those deemed as having ‘high’ fluoride exposure (defined in various ways). Lin *et al.* (1991) (27) found individuals residing in high fluoride areas (defined as areas with an average fluoride concentration of 0.88 parts per million (ppm) in drinking water) had significantly higher TSH levels than those residing in low fluoride areas (average fluoride concentration of 0.34 ppm in drinking water) (p<0.01). Michael *et al.* (1996) (28) found significant increases in T4 level in population with high fluoride exposure. Xiang 2009 (24) reported the high fluoride exposure can cause the thyroid functional abnormalities. The reason for this contradiction may be the higher water fluoride levels of study location (2.36 to 14 ppm) in comparison to ours 1.4ppm. Also, nutritional status and normal iodine consumption were not considered.

In present study normal nutritional states and optimal iodine intakes were considered as selection criteria because studies reported greater possibility of endemic goiter in iodine-deficient communities especially in low socioeconomic children with a poor nutritional status (Jooste *et al.*, 1997) (29). Dietary iodine intake has proved to influence the epidemiology of thyroid dysfunction. Iodine forms an essential component of the hormones produced by the thyroid gland (30). In areas where the daily iodine intake is <50 μg, goiter is reported to be endemic with the prevalence of 80% and when the daily intake is <25 μg, congenital hypothyroidism is observed.

According to the World Health Organization, the simplest, most effective and inexpensive preventive method is the consumption of iodized salt. In India to combat iodine deficiency disorders it has been more than three decades since universal salt iodization program was introduced. India is undergoing a transition from iodine deficient to iodine sufficient state (31).

A study by Jooste PL in 1999 (22) reported fluoride at 1.7ppm and above may behave as a goitrogen in optimal iodine population. Our study results showed non-significant association between thyroid function and fluoride up to 1.4ppm.

Relating dental fluorosis to thyroid dysfunction Schuld 2005 (32) reported tooth and thyroid gland develops almost at same age so fluorosis and thyroid dysfunction may be correlated. Supporting above hypothesis study by Xiang 2009(24) reported the different severity degree of Dental Fluorosis may be related to significant deviation in the serum levels of thyroid hormone. In contradiction present study results shown non-significant association between dental fluorosis to thyroid function. In accordance with our findings, a study by Hosur et al (2012) (33) reportedthat childrenwith dental fluorosis had normal thyroid hormone levels.

Mean serum fluoride levels of childrenacross the groups showed an increasing trend from group I to III. Similar findings were noted in study by Susheela *et al.*, (2005)(2) Singh N *et al.*, (2014) (34).

To carry out such a project, selection of study area plays a vital role. The need was an area with natural, independent, unaltered near optimum ground water fluoride. So we selected an area located at 12.30°N 74.65°E which has an average altitude of 770 metres (2,526 ft). It is spread across an area of 128.42 km2 (50 sq mi). It has a tropical savanna climate designated “Aw” under the Köppen climate classification with average annual rainfall is 804.2 mm (31.7 in). In entire the study area groundwater is the major source for drinking water as centralized surface water supply is only restricted to the city limits.

In the present study, the final villages for sampling were selected based on drinking water fluoride levels estimated as per APHA (1998) guidelines using ISE method. Different methods and instruments have been used to estimate fluoride levels in drinking water such as Ion Chromatography (Cochrane *et al.*, 2014), (35) Colorimetric (Gail *et al.*, 1987) (36) Potentiometric (Egorov *et al.*, 2008) (37) and Spectrophotometric method (Zaher and Sameer, 2012) (38). We preferred ISE method because of its advantages like accuracy, speed, economy and sensitivity in comparison with other methods like UV/VIS spectroscopy.

In present study the level of serum T3, T4 were determined by Competitive Chemi Luminescent Immuno Assay(CLIA) and TSH was determined by Ultra sensitive sandwich chemiluminescentimmuno assay. In the presence of complimentary antigen and antibody, the paratope of the antibody binds to the epitope of the antigen to form an antigen-antibody complex or an immune complex. Estimating the levels of such immune complex by the use of labeled antibodies forms the basis of CLIA. It involves use of stationary solid particles coated either with the antigen or antibody of interest for post incubation intact immune complex formation which generates light and the intensity of which is directly proportional to the amount of labeled complexes present and which indirectly aids in quantification of the analyte of interest. The intensity of light is measured in terms of Relative Light Units (RLU) .The main advantage of this technology includes sensitivity and its ability to remain stable for background signals. Also, the analyzers working under this principle are simple in design and operation.

Dietary fluoride consumption strongly influences total fluoride intake. However we have only considered drinking water fluoride level of children and this is one of the limitations of present study.

## Conclusions

Within the limitations of the present study long term intake of naturally fluoridated drinking water (Within the range of 0.02 -1.4 ppm) doesn’t seem to have any effect on the thyroid function in the children with normal nutritional status and optimal iodine intake.
